# Supplementary material for: Ten simple rules for making research software more robust
Source: PLoS Comput Biol. 2017 Apr 13;13(4):e1005412. doi: 10.1371/journal.pcbi.1005412 (PMC5390961; doi:10.1371/journal.pcbi.1005412)
Supplement: S1 Checklist — A checklist summarizing these ten simple rules to apply to your own software. (PDF) [file pcbi.1005412.s001.pdf]

Ten Simple Rules for Making Research Software More Robust: The Checklist

- ☐ **Use version control.**
  - ☐ Put everything that you write or make into version control as soon as it is created.
  - ☐ Use a feature branch workflow.
- ☐ **Document your code and usage.**
  - ☐ Write a good README file.
  - ☐ Print usage information.
- ☐ **Make common operations easy to control.**
  - ☐ Allow the most commonly changed parameters to be configured from the command line.
  - ☐ Check that all input values are in a reasonable range at startup.
  - ☐ Choose reasonable defaults where they exist.
  - ☐ Set no defaults at all when there aren't any reasonable ones.
- ☐ **Version your releases.**
  - ☐ Increment your version number every time you release your software to other people.
  - ☐ Make the version of your software easily available by supplying `--version` or `-v` on the command line.
  - ☐ Include the version number in in all of the program's output.
  - ☐ Ensure that old released versions continue to be available.
- ☐ **Reuse software (within reason).**
  - ☐ Make sure that you really need the auxiliary program.
  - ☐ Ensure the appropriate software and version is available.
  - ☐ Ensure that reused software is robust.
- ☐ **Rely on build tools and package managers for installation.**
  - ☐ Document all dependencies in a machine-readable form.
  - ☐ Avoid depending on scripts and tools which are not available as packages.
- ☐ **Do not require root or other special privileges to install or run.**
  - ☐ Do not require root privileges to set up or use packages.
  - ☐ Allow packages to be installed in an arbitrary location.
  - ☐ Ask another person to try and build your software before releasing it.
- ☐ **Eliminate hard-coded paths.**
  - ☐ Set the names and locations of input and output files as command-line parameters.
  - ☐ Do not require users to navigate to a particular directory to do their work.
- ☐ **Include a small test set that can be run to ensure the software is actually working.**
  - ☐ Make the tests easy to find and run.
  - ☐ Make the test script's output easy to interpret.
- ☐ **Produce identical results when given identical inputs.**
  - ☐ Echo all parameters and software versions to standard out or a log file alongside the results.
  - ☐ Produce the same results each time the same version of the program is run with the same inputs.
  - ☐ Allow the user to optionally provide the random seed as an input parameter.
  - ☐ Make sure acceptable tolerances are known and detailed in documentation and tests.
